# High Performance of Supercapacitor from PEDOT:PSS Electrode and Redox Iodide Ion Electrolyte

**DOI:** 10.3390/nano8050335

**Published:** 2018-05-16

**Authors:** Xing Gao, Lei Zu, Xiaomin Cai, Ce Li, Huiqin Lian, Yang Liu, Xiaodong Wang, Xiuguo Cui

**Affiliations:** 1 School of Material Science and Engineering, Beijing Institute of Petrochemical Technology, Beijing 102617, China; gaoxing@bipt.edu.cn (X.G.); zulei@bipt.edu.cn (L.Z.); 13522433853@163.com (X.C.); lice0502@163.com (C.L.); yang.liu@bipt.edu.cn (Y.L.); 2 State Key Laboratory of Organic-Inorganic Composites, Beijing University of Chemical Technology, Beijing 100029, China; wangxd@mail.buct.edu.cn; 3 Beijing Key Laboratory of Specialty Elastomer Composite Materials, Beijing Institute of Petrochemical Technology, Beijing 102617, China

**Keywords:** PEDOT:PSS, catalysis, redox electrolyte, stability

## Abstract

Insufficient energy density and poor cyclic stability is still challenge for conductive polymer-based supercapacitor. Herein, high performance electrochemical system has been assembled by combining poly (3,4-ethylenedioxythiophene) (PEDOT):poly (styrene sulfonate) (PSS) redox electrode and potassium iodide redox electrolyte, which provide the maximum specific capacity of 51.3 mAh/g and the retention of specific capacity of 87.6% after 3000 cycles due to the synergic effect through a simultaneous redox reaction both in electrode and electrolyte, as well as the catalytic activity for reduction of triiodide of the PEDOT:PSS.

## 1. Introduction

As a promising and versatile energy storage device, the supercapacitor has a the ability to meet the demands of various application fields such as hybrid electric vehicles, portable electronic and uninterruptable power [1]. Except for its insufficient energy density, supercapacitor usually can provide a higher power density and longer the cyclic life than that of the secondary ion batteries. Thus, significant explorations and developments have been devoted to enhance the energy density through increasing either or both the capacitance and the cell voltage [2]. Recently, an innovative strategy has been researched to increase the specific capacitance by introducing a redox-active compound of the electrolyte in carbon based electrochemical supercapacitors [3]. After that, all kinds of redox active electrolytes including organic and inorganic active mediators have been employed for high capacitance supercapacitors [4,5,6].

In the above-mentioned supercapacitor, which composed of carbonaceous electrode and redox aqueous electrolyte, a striking high capacitance was obtained only at positive electrode, but a relative low capacitance was still showed in the two-electrode system. This is because the total capacitance of an electrochemical supercapacitor is largely depended on the electrode with low capacitance [7]. On the other hand, since the capacitance of supercapacitor is resulted from both of the low electric double layers capacitance and high pseudo capacitance of redox active electrolyte, the electrochemical combination of carbon electrode and active electrolyte is unsatisfactory match [8]. In order to improve the high performance of supercapacitor, it is worth to explore well match between electrode and electrolyte as well as synergetic effect each other, for instance, electrochemical combinations of redox electrode and redox electrolyte. Interestingly, some new combination such as polyaniline-graphene/redox additive of hydroquinone, manganese dioxide/redox electrolyte of p-phenylenediamine were began to be reported [9,10]. In our previous works, we have systematically studied various electrochemical combinations which consisted of a redox electrode and a redox additive electrolyte, including polyaniline-multiwall carbon nanotube composite electrode/potassium iodide redox electrolyte [7], mesoporous silica/polyaniline composite electrode-KI in the sulfuric acid aqueous electrolyte [11,12], polyaniline-carbon nanotube composite electrode/hydroquinone redox electrolyte [13], mesoporous manganese dioxide/silver nanowire composite electrode-KI in the sodium sulfate aqueous electrolyte [14], polyaniline-Nano nickel oxide/KI [15] and manganese dioxide/polyaniline composite electrode-KI electrolyte [16].

Among these various systems, the polyaniline-based composite electrode-KI electrolyte electrochemical system has presented a maximum capacitance of 3331 F/g at 1 A/g of current density, and the retention rate of capacitance is 78% after 1000 cycles. However, the capacitance value is only 726 F/g in the two-electrode system [8]. Obviously, the retention of capacitance is far from satisfactory when we compare the cyclic stability of the pseudo capacitor with that of the electric double layers capacitors. It can be ascribed to an abnormal phenomenon to the electrochemical degradation of polyaniline [17]. Unlike polyaniline, whose intrinsic defect still is a challenge for supercapacitors, PEDOT:PSS is a promising electrode material for its high electrical conductivity, good environmental stability [18]. A typical example is polythiophene and the active materials, which provided high specific capacity of 40 mAh/g and outstanding cycle stability where its high capacity keeps in 500 cycles [18,19].

In this paper, we report a new electrochemical supercapacitor that made of a PEDOT:PSS redox composite electrode and KI redox aqueous electrolyte, which can provide an electrochemical synergic effect through a simultaneous redox reaction in both of the electrode and electrolyte. It is notable to that the redox of iodine ion can be promoted by catalyst effect of PEDOT:PSS. By virtue of this merit, the supercapacitor exhibited the maximum specific capacity of 51.3 mAh/g, which is 1.28 times of the correspondent electrochemical systems [18]. For the cycle stability, the retention of specific capacity is 87.6% after 3000 cycles in our two-electrode system, which more excellent than the past reports.

## 2. Materials and Methods

### 2.1. Materials

Multi-walled carbon nanotubes (MWCNTs) were obtained from CNano Technology Co., Ltd., Beijing, China. Active carbon (AC) was bought from Tianjin Saibo (YP-50F, Tianjin, China). PEDOT:PSS aqueous dispersion (1.3 wt %) was supplied by Baytron (HC Starck’s Clevios PH1000, Shanghai, China) without further treatment. The Poly (vinyl alcohol) (PVA) 1788 with solution of 87.0–89.0% (mol/mol) was purchased from Aladdin Co., Ltd., Shanghai, China.

### 2.2. Modification of MWCNTs

The method of the modified MWCNTs was based on previously reported [20]. Firstly, 0.2 g MWCNTs were added into a mixture of H_2_SO_4_ (3 mol/L) and HNO_3_ (3 mol/L) (3:1) in the round flask and sonicated for 30 min. Secondly, it was heated to 120 °C and refluxed for 12 h. And then, the MWCNTs was filtrated and washed until neutral. Finally, the sample was dried at 60 °C under vacuum for 24 h.

### 2.3. Preparation of PEDOT:PSS/MWCNTs Composite Electrode

The preparation procedure of PEDOT:PSS/MWCNTs electrodes is described as follows. At first, 80% PEDOT:PSS and 10% MWCNTs were mixed under sonicated for 1 h to obtain stable dispersion. And then, 10% PVA used as bonding agent was put into the dispersion under the condition of vigorous stirring for 1 h. The mass of the electrode material is controlled in 4 mg. Coating PEDOT:PSS/MWCNTs on stainless steel was performed by a “dip and dry coating” technique [21,22]. The stainless steel was immersed in solution of PEDOT:PSS/MWCNTs for 9 s and then dried under the infra-red lamp for 8 min. The process was repeated twice to increase the PEDOT:PSS/MWCNTs loading in the stainless steel and make sure a consistent coating. The net mass of the PEDOT:PSS/MWCNTs was obtained by calculating and weighing.

### 2.4. Characterization

The morphology of the MWCNTs nanowire and PEDOT:PSS/MWCNTs nanowire were observed on scanning electron microscopy (SEM) of COXEM-20 microscope (COXEM, Daejeon, Korea) at 20 KV. The structure of the PEDOT:PSS/MWCNTs thin films was characterized by X-ray diffractometer (XRD) with a Bruker D8 diffractometer (BRUKER AXS, Berlin, Germany) using a Cu Kα source operated at 40 kV.

The electrochemical measurements of Cyclic voltammetry (CV), galvanostatic charge–discharge (GCD), and electrochemical impedance spectroscopy experiments (EIS) were tested by a standard three-electrode system using a CHI 660D electrochemical workstation (Chenhua Co., Shanghai, China). A saturated calomel electrode (SCE) was used as the reference electrode and used a Pt foil electrode (about 1 cm^2^) as the counter electrode. The electrolyte was composed of 1 M H_2_SO_4_ and 0.02, 0.05, 0.1, 0.2 M KI, respectively.

The specific capacitance (Cs, F/g) of the electrodes was calculated according to the Equations (1)

(1) $$C=\frac{I\times \Delta t}{m\times \Delta v}$$

Cycling performance of the PEDOT:PSS/MWCNTs was investigated by using LANHE CT2001A (Landian Co., Wuhan, China) at a current density of 1 A/g.

## 3. Results and Discussion

### 3.1. Properties of the PEDOT:PSS/MWCNTs Films

**Figure 1:**
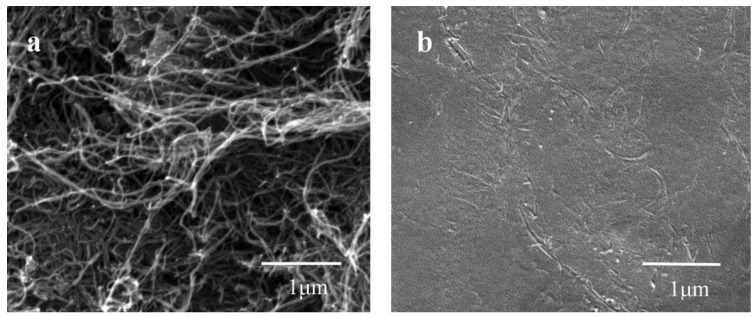
(**a**) SEM of multi-walled carbon nanotubes (MWCNTs); (**b**) Composite of poly (3,4-ethylenedioxythiophene):poly (styrene sulfonate) (PEDOT:PSS)/MWCNTs.

The modified MWCNT with hydrophilic group is well dispersed in the PEDOT:PSS solution (Figure 1a). Introducing MWCNTs into the PEDOT:PSS can improve the conductivity and stability of the PEDOT:PSS/MWCNTs films, Due to the modified MWCNTs could be dispersed uniformly throughout the polymer matrix, as shown in Figure 1b.

**Figure 2:**
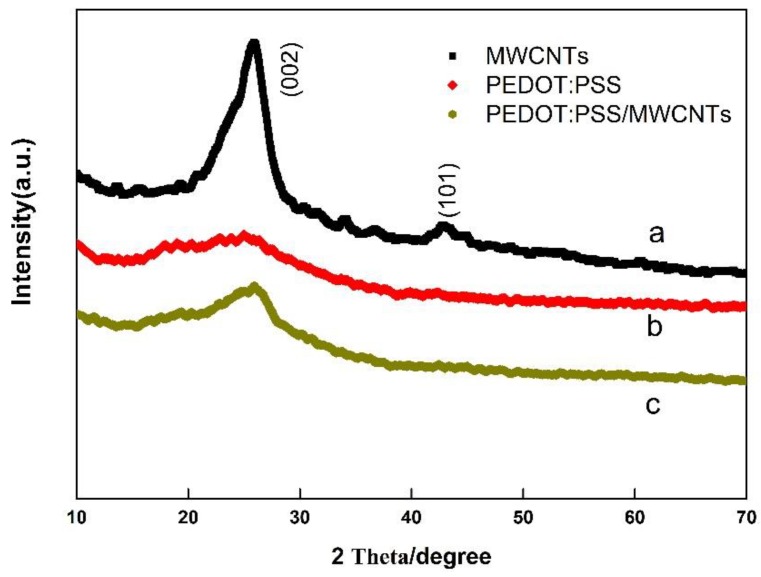
XRD patterns of MWCNTs (**a**), PEDOT:PSS (**b**) and PEDOT:PSS/MWCNTs (**c**).

To further explore the micro-structure of PEDOT:PSS/MWCNTs thin films, the XRD analysis of MWCNTs, PEDOT:PSS, and PEDOT:PSS/MWCNTs were investigated. In Figure 2a, the characteristic peaks of MWCNTs at 25.9° and 42.9° could be indexed as (002)and (101) reflection, respectively [23]. As displayed in Figure 2b, pure PEDOT: PSS has not any characteristic peak except for the peak at 25.9°, manifesting the amorphous of the polymeric material [24]. The intensity of all diffraction peaks of MWCNTs decreased, suggesting a well-dispersion of MWCNTs in the composite film.

### 3.2. Electrochemical Properties of the PEDOT:PSS/MWCNTs Electrodes

**Figure 3:**
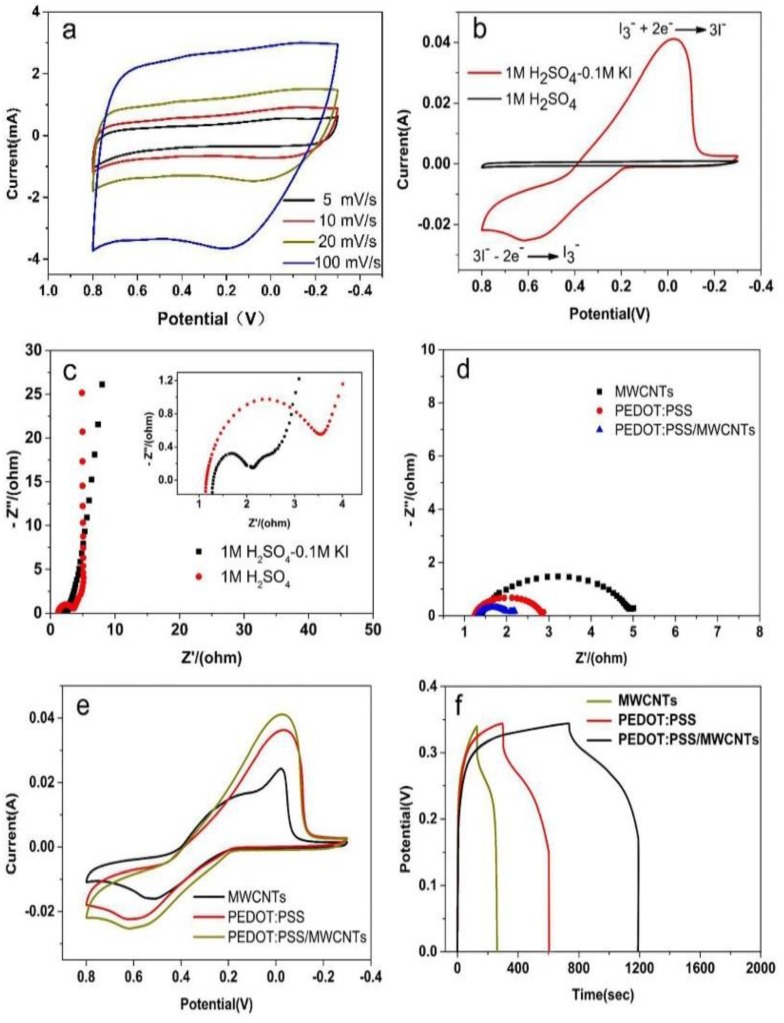
Electrochemical experiments data of PEDOT:PSS/MWCNTs electrodes in 1 M H_2_SO_4_ and 1 M H_2_SO_4_-0.1 M KI electrolytes. (**a**) Cyclic voltammetry (CV) curves of the PEDOT:PSS/MWCNTs electrode at scan rates of 5–100 mv/s in the 1 M H_2_SO_4_ electrolytes. (**b**) CV curves of the PEDOT:PSS/MWCNTs at san rates of 10 mv/s. (**c**) Nyquist plot for the PEDOT:PSS/MWCNTs electrode in different electrolytes (The inset, low-frequency region in the top right). (**d**) Nyquist plots of charge-transfer resistance (RCT) with a variety of materials. (**e**) CV curves at a scan rate of 10 mv/s. (**f**) GCD of MWCNTs, PEDOT:PSS and PEDOT:PSS/MWCNTs.

**Figure 4:**
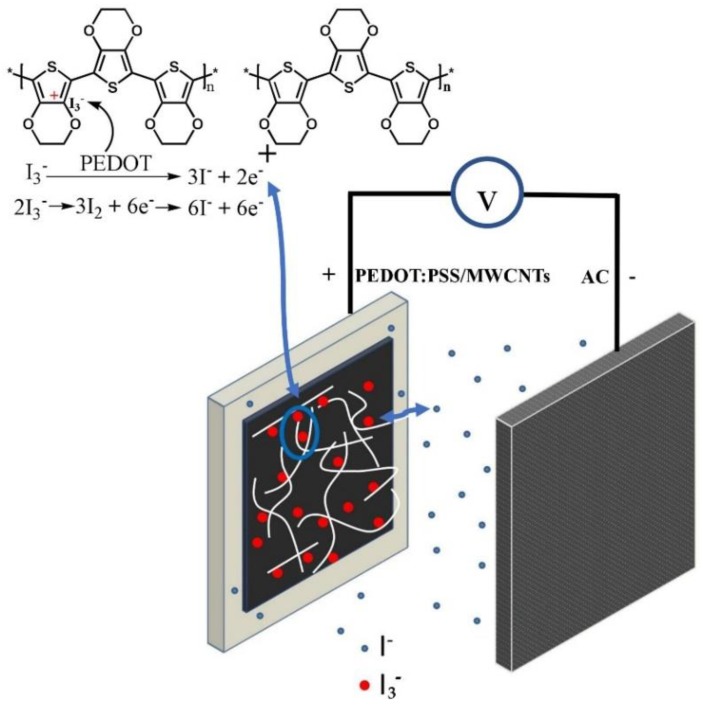
Schematics of the chemistry of the supercapacitor. The inset in the upper left corner shows the mechanism of triiodide reduction.

The electrochemical properties of electrodes materials were investigated by using a three-electrode configuration. The quasi-rectangular shaped CV curves of the PEDOT:PSS/MWCNTs (Figure 3a) were measured in 1 M H_2_SO_4_ (at a scan rate of 5–100 mv/s, the voltage window of −0.3~0.8 V). The above results showed that the response of the PEDOT:PSS/MWCNTs electrode to redox reactions is so quick and its resistance is rather low [25], which is consistent with the previous reports on conductive polymer-based composites. Figure 3b is the CV curves of PEDOT:PSS/MWCNTs tested in the two electrolytes. It was obviously observed that PEDOT:PSS/MWCNTs had a couple of sensitive redox peaks at −0.02 and 0.62 V in the 1 M H_2_SO_4_-0.1 M KI, additionally, CV curves of the PEDOT:PSS/MWCNTs in the 1 M H_2_SO_4_-0.1 M KI covered more current area than that of the 1 M H_2_SO_4_. The reasons are as follows: (1) Iodide ions redox reactions between different valence states occurred inside or on the surface of PEDOT:PSS/MWCNTs electrode material [26]; (2) The reduction of $I_{3}^{-}$/3$I^{-}$ is catalyzed by the chains of PEDOT:PSS [27,28], which results in the enhancement of the specific capacity of the electrode material. As is shown in Figure 4, $I^{-}$ ions are oxidized to $I_{3}^{-}$ ions, and then $I_{3}^{-}$ ions migrate to the PEDOT:PSS/MWCNTs electrode. In fact, $I_{3}^{-}$ ions may be reduced to I_2_ [29]. The reduce reaction from PEDOT catalytic effects can be avoided. Thus, a couple of the asymmetric redox peaks was observed and the intensity of reduction peak is stronger than oxidation peak.

Figure 3c showed that the addition of KI results in the reduction of the charge-transfer resistances (RCT) of PEDOT:PSS/MWCNTs from 2.46 (1 M H_2_SO_4_) to 0.81 Ω cm^−2^ (1 M H_2_SO_4_-0.1 M KI). As was shown in Figure 3d, Nyquist plots for charge-transfer resistance (RCT) are 0.81, 1.53 and 3.55 Ω cm^−2^ in 1 M H_2_SO_4_-0.1 M KI, respectively. The catalytic activity of MWCNTs for reduction of $I_{3}^{-}$ can be characterized by using EIS [30]. The activity of the triiodide reduction become more and more weaker with increasing the electron transfer resistance. Therefore, the PEDOT:PSS has a catalytic activity for reduction of triiodide.

Figure 3e also revealed PEDOT had catalyzed tri-iodide reduced to iodide since the intensity of reduction peaks of MWCNTs was obviously smaller than PEDOT:PSS or PEDOT:PSS/MWCNTs. The GCD curves of PEDOT:PSS/MWCNTs, PEDOT:PSS and MWCNTs electrodes within a voltage window of 0 to 0.35 V and at a current density of 1 A/g were shown in Figure 3f. The profiles displayed that the PEDOT:PSS/MWCNTs exhibited an excellent discharge property among these three materials, consistent with the result of the CV curves (Figure 3e). Owing to the linear discharge curves, the special capacitances were calculated with Equation (1) using discharge time. The specific capacitance of PEDOT:PSS/MWCNTs is 1314.24 F/g, which is 1.5 times larger than that of the PEDOT:PSS (863.37 F/g) and almost 3.5 times greater than that of the MWCNTs (392.35 F/g).

**Figure 5:**
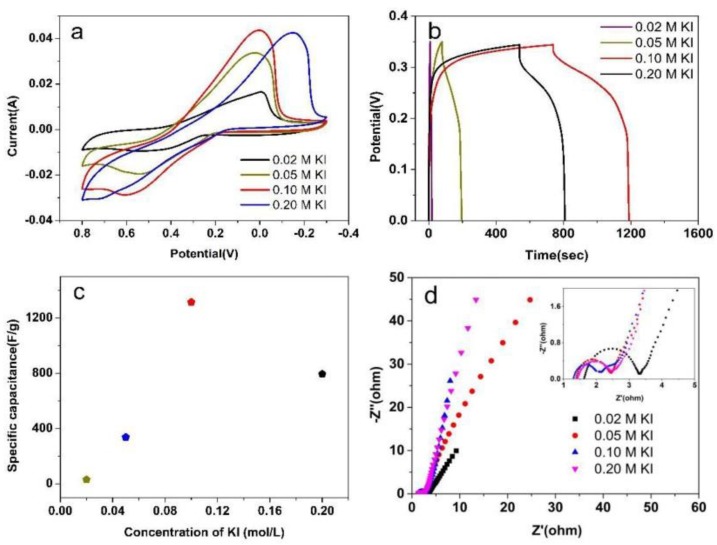
(**a**) CV curves recorded at 20 mV/s. (**b**) Galvanostatic charge–discharge (GCD) profiles at 1 A/g. (**c**) Calculated specific capacitance for 1 A/g at different concentration of KI. (**d**) Nyquist plot of PEDOT:PSS/MWCNTs electrodes (The inset showed high-frequency region in the top right).

Moreover, the electrochemical performances were studied at different concentrations of KI. Figure 5a displayed the CV curves of the four concentrations of KI measured at a scan rate of 20 mV/s with a potential range from −0.3 to 0.8 V. The CV curves showed an ideal oxidation and reduction peak at 0.5–0.7 V and −0.2–0 V. Furthermore, the CV curves showed a lager integrated area in 1 M H_2_SO_4_-0.1 M KI electrolyte than other concentrations of KI, indicating the PEDOT:PSS/MWCNTs had a largest specific capacitance at this concentration. Iodide is a very appealing candidate, it ensured the ionic conductivity and supplied the pseudo-capacitive effects [31], which have provided great advantages because of its inertness, high conductivity and environmental friendliness.

The ultimate and reasonable technique for capacitance measurements is GCD analysis, therefore the GCD test for all concentrations of KI were carried out at 1 A/g in a potential window from 0 to 0.35 V (Figure 5b). As expected, the discharge duration of PEDOT:PSS/MWCNTs at 1 M H_2_SO_4_-0.1 M KI is much longer than that of the other concentrations, meaning the highest specific capacitance, which is consistent with the above CV results. Based on Equation (1), the specific capacitance of PEDOT:PSS/MWCNTs at different concentration of KI (0.02, 0.05, 0.10 and 0.20 mol/L) was calculated at 1 A/g (Figure 5c). The specific capacitance of the PEDOT:PSS/MWCNTs was estimated to be 1314.24 F/g at 1 M H_2_SO_4_-0.1 M KI. Therefore, the electrochemical performance is inhibited since there may exist some disturbing factor if the concentration of iodide ions exceeds the critical point. The process of the reduction of $I_{3}^{-}$/3I^−^ may be restrained with high-concentrated iodide ions, and then affect the specific capacitance.

The PEDOT:PSS/MWCNTs electrode was further characterized by using EIS at different concentrations of KI shown in Figure 5d and the inset showed magnified portion of EIS in the high-frequency region. These plots are composed of high frequency region and low frequency. In the high frequency intercept of the real axis, an internal resistance (Rs) can be observed and the charge-transfer resistance (Rct) are calculated from the diameters of semicircle in the medium-to-low frequency region [32].

**Figure 6:**
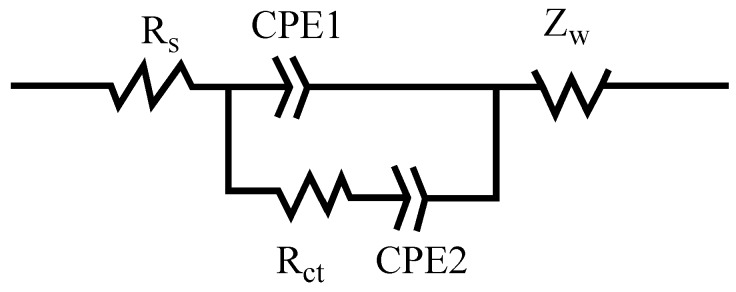
The equivalent circuit for PEDOT:PSS/MWCNTs at different concentrations of KI with 1 M H_2_SO_4_ electrolytes.

Figure 6 showed an equivalent circuit for PEDOT:PSS/MWCNTs at different concentrations of KI with 1 M H_2_SO_4_ electrolytes. Table 1 displayed the best fitness EIS values. The results of standard deviation were simulated by software of ZSimpWin. The Rs values of different concentrations are almost the same. Thus, Rct is major difference at different KI concentration with 1 M H_2_SO_4_ comparing Rs. It is obvious to see that the Rct values decrease with increasing the concentration of KI until up to 0.1 mol/L. The charge transfer resistance Rct is found to be 0.81 Ω cm^−2^ for concentration of 0.1 M KI. The charge transfer resistance Rct is associated with the electron transfer process and is inversely related to the exchange current density [33,34]. Therefore, if Rct is smaller, it means a facile interfacial electron transfer process and thus a higher specific capacitance [34].

**Figure 7:**
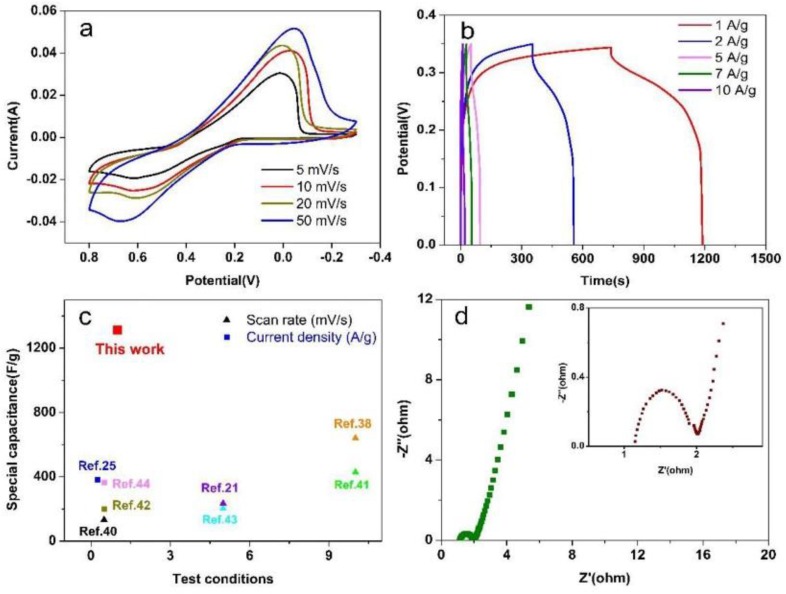
Electrochemical analysis and comparison investigated at 1 M H_2_SO_4_-0.1 M KI. (**a**) CV curves of the PEDOT:PSS/MWCNTs at different scan rates. (**b**) GCD profiles of the PEDOT:PSS/MWCNTs electrode at different current densities. (**c**) Comparison of specific capacitance of PEDOT:PSS/MWCNTs with reported values. (**d**) Nyquist plot of PEDOT:PSS/MWCNTs electrodes (The inset showed high-frequency region in the top right).

Figure 7a presented the CV measurements of the PEDOT:PSS/MWCNTs electrode at a scan rate of 5, 10, 20 and 50 mV/s, respectively, in the 1 M H_2_SO_4_-0.1 M KI. The CV curves indicate that as the scan rate increasing, the specific capacitance decreases because the oxidation-reduction process did not react fully in time at high scan rate. The increase in the scan rate directly affects the diffusion of $I_{3}^{-}$ ions into the PEDOT:PSS and just infiltrate some large pores [35], which cannot enter into the PEDOT:PSS/MWCNTs but the outer surface at high scan rate [36,37,38]. When increasing the scan rate, a pair of positive shift of the oxidation and negative shift of the reduction peak can be detected, which is mainly owing to the resistance of the electrode [36,39].

The GCD curves of PEDOT:PSS/MWCNTs electrode tested at different current densities (1 M H_2_SO_4_-0.1 M KI electrolyte) were shown in Figure 7b, which manifested that the specific capacitance decreased with increasing of current density. The specific capacitance of the PEDOT:PSS/MWCNTs electrode was estimated to be 1314.4 F/g at 1 A/g. It still achieves 291.43 F/g even at 10 A/g, exhibiting a good high-rate discharge ability. Comparing specific capacitance of super-capacitors with reported values in Figure 7c, the PEDOT:PSS/MWCNTs electrode achieved a specific capacity of 1314.24 F/g (1 A/g) in 1 M H_2_SO_4_-0.1 M KI, which is better than that of some similar electrodes based on conducting polymers and or carbon materials, including PEDOT-PSS/SWCNT (single-walled carbon nanotubes) [40], PEDOT:PSS/MWCNTs [21], active carbon/PEDOT:PSS [38], PEDOT:PSS/MWNT/MnO_2_ [41], PEDOT:MWCNT:PTS [42], PEDOT/HT-CFC (hydrothermal carbon fibre cloth) [43], PEDOT/Fc (ferrocene) [44], PEDOT:PSS/MWCNT [25]. Figure 7d showed Nyquist plot of PEDOT:PSS/MWCNTs electrodes in 1 M H_2_SO_4_-1 M KI. 

**Figure 8:**
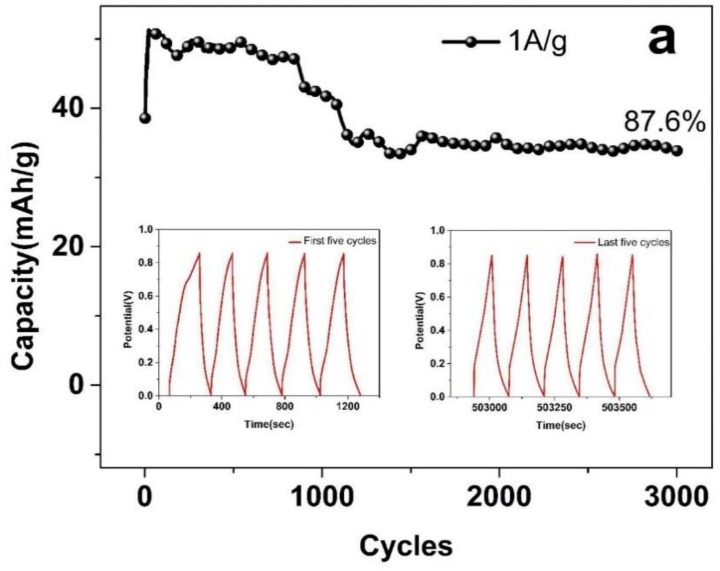

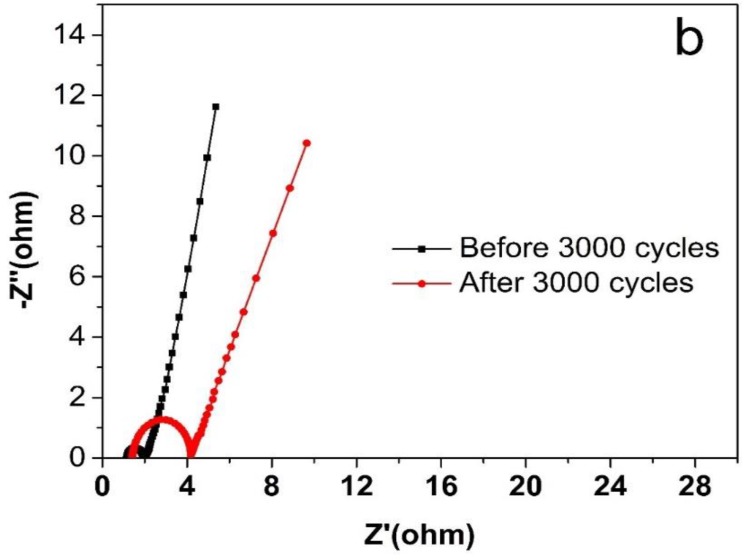
(**a**) Cycle life performance of the PEDOT:PSS/MWCNTs electrodes at a current density of 1 A/g with two-electrode asymmetric electrochemical system. (**b**) Nyquist plot of PEDOT:PSS/MWCNTs electrodes before and after long-term stability test.

It is worth noting that the cycle performances are crucial for electrochemical supercapacitors. The cycle life performance of the PEDOT:PSS/MWCNTs electrodes is investigated by a continuous cycling test over 3000 cycles at 1 A/g. As shown in Figure 8a, it is obviously to find that the capacity for the supercapacitor increases from 38.5 to 51.3 mAh/g in the initial 50 cycles. The mass of calculating the capacity is the total mass of positive electrode (4.2 mg). The inset shows charge/discharge curves of the first five and last five charge–discharge cycles within a voltage window of 0–0.85 V. The capacity increasing at the beginning can be attributed to self-activation process of the device [37]. The ion adsorption and desorption between electrodes and electrolyte will be more sufficient contacts after some circulations at the start, which increase active sites in the electrode material and capacity [45]. After 3000 cycles, the retained capacity (33.8 mAh/g) is still 87.6% of the initial value (38.5 mAh/g), which is higher than that of Ag–PEDOT:PSS–MnO_2_ (86.8% after 2000 cycles) [46], PEDOT/GO (graphene oxide) (86% after 1000 cycles) [47], PEDOT-NWs/CC (nanowires/carbon cloth) (70% after 1000 cycles) [48]. The good cycling stability of the asymmetric supercapacitor also can be displayed by the charge–discharge curve of the PEDOT:PSS/MWCNTs electrode (see inset in Figure 8a) within a voltage window of 0–0.85 V. It is obvious to see that each charge and discharge curves are consistent after 2950 cycles, implying no evident structural change of the PEDOT:PSS/MWCNTs electrode during the charge–discharge processes and cycling stability. Furthermore, a decrease in capacity was observed after 3000 cycles, which can be ascribed to the increase in charge transfer resistance [49]. Figure 8b shows Nyquist plot of PEDOT:PSS/MWCNTs electrodes before and after long-term stability test, the charge transfer resistance increasing from 0.84 to 2.78 Ω cm^−2^.

## 4. Conclusions

In summary, the multi-synergetic effects have been harvested by a series of promoting factors including a new electrochemical combination of PEDOT:PSS redox electrode and KI redox electrolyte, as well as the catalyzing of PEDOT for tri-iodide. Based on the investigation of concentration of KI for performance of electrodes in three-electrode system, the results exhibited that the optimum concentration of KI is 0.1 M, the maximum specific capacitance of PEDOT:PSS/MWCNTs composite of 1314.24 F/g has been achieved at 1 A/g in 1 M H_2_SO_4_-0.1 M KI. Significantly, the capacity of PEDOT:PSS/MWCNTs/KI conjugation in two-electrode system provides a high capacity of 51.3 mAh/g and a good cycling life of 87.6% retention and small electric resistance of 2.78 Ω cm^−2^ after 3000 cycles, which is attributed to the stable conductive network consisted of PEDOT and carbon nanotube.

## Figures and Tables

**Table 1:** The standard deviation, electrolyte resistance and charge-transfer resistance at different KI concentration with 1 M H_2_SO_4_.

KI (mol/L)	Rs (Ω **cm^−2^)**	Rct (Ω **cm^−2^**)	Rct Standard Deviation (%)
0	1.14	2.46	5.1
0.02	1.65	1.68	2.109
0.05	1.37	1.09	2.55
0.10	1.30	0.81	2.555
0.20	1.44	1.03	3.169
0.10 (stability test)	1.403	2.78	0.6711

## Abbreviations

PEDOT:PSS	poly(3,4-ethylenedioxythiophene) (PEDOT):poly (styrene sulfonate) (PSS)
MWCNTs	Multi-walled carbon nanotubes
AC	Active carbon
SEM	Scanning electron microscopy
XRD	X-ray diffractometer
PVA	Polyvinyl alcohol
